# Prevalence, Awareness, Treatment, and Control of Hypertension and Diabetes: Results From Two State-Wide STEPS Survey in Punjab and Haryana, India

**DOI:** 10.3389/fpubh.2022.768471

**Published:** 2022-03-21

**Authors:** J. S. Thakur, Ria Nangia

**Affiliations:** Department of Community Medicine and School of Public Health, Post Graduate Institute of Medical Education and Research, Chandigarh, India

**Keywords:** awareness, treatment, NCDs, hypertension, diabetes, Punjab, Haryana, STEPS survey

## Abstract

**Background:**

India which is home to more than one sixth of the world's population, accounts for more than two thirds of total deaths due to non-communicable diseases (NCD). Out of this, hypertension and diabetes are the most common NCDs. Awareness, treatment, and control of hypertension and diabetes remains a major challenge despite various national programs being run to curb the rising burden NCDs. In order to fill the knowledge gap, awareness, treatment, and control of diabetes and hypertension were studied by using data from the STEPS survey among the adult population in two major northern Indian states of Punjab and Haryana.

**Methods:**

Two state-wide NCD risk factors surveys were conducted using WHO STEPS methodology among 5,127 individuals in Punjab and 5,078 individuals in Haryana aged 18–69 years in the year 2014–15 and 2016–18. Standardized questionnaire was used to determine the behavioral risk factors in step one followed by anthropometric measurements for physical risk factors in step two and in the third step serum and urine samples were collected for biochemical risk factors.

**Results:**

The prevalence of hypertension in Punjab was 40.1% while that in Haryana was 26.2%. In Punjab, only 48.3% of the hypertensive were aware of their condition, 30.9% were on treatment while only 18.3% of the cases were controlled. While in Haryana 33.4% of the respondents were aware of their condition, 26.3% are on treatment while only 12% of the cases were controlled. Similarly, the prevalence of diabetes was 14.3 and 15.1% in Punjab and Haryana, respectively. In Punjab 34.2% of diabetics were aware of their condition, 28.2% were on treatment while only 14.2% of the cases were controlled. The awareness and control rates in Haryana were similar to that in Punjab. 29.5% of the respondents were aware of their condition, 22.4% were on treatment while only 13.8% of the cases of diabetes were controlled. Family history of diabetes and hypertension was found to be associated with higher odds of being aware, on treatment and controlled blood glucose and blood pressure levels in both Punjab and Haryana.

**Discussion:**

Hypertension and diabetes are a major public health problem in Punjab and Haryana and awareness, treatment and control rates are low which require specific interventions with a focus on access to treatment, regular follow up for better control. There is an urgent need to effectively implement the existing national NCD programmes in these states in India.

## Introduction

The global burden of non-communicable diseases (NCDs) has a disproportionate impact on low- and middle-income countries (LMICs). An estimated 41 million people worldwide died from NCDs in 2016, of which more than 78% occurred in low- and middle-income countries (1, 2). The burden of hypertension and diabetes has steadily increased over the past 50 years globally, with India contributing a major part of the global burden (3). In India, almost 5.8 million people die every year from NCDs, mainly heart and lung diseases, stroke, cancer and diabetes, meaning a quarter of Indians are at risk of dying from a NCD before the age of 70 years. India has more than one sixth of the world's population, and accounts for more than two thirds of all deaths from non-NCDs (4). Among them, hypertension and diabetes are the most NCDs. The recent epidemiological evidence shows that the incidence and prevalence of the disease in both urban and rural India is on the rise (5, 6).

Early diagnosis and control of hypertension and diabetes can reduce premature mortality and disability (7). However, studies suggest that around half of the population living with hypertension have not been diagnosed while one third of the population with diabetes are unaware of their condition (8, 9). The National Health Policy 2017 of India also aims to increase screening and treatment of 80% of people with diabetes and hypertension to reduce premature deaths from diabetes by 25% by 2025 (10).

Several studies report that patients in rural and urban areas around the world have low levels of awareness of hypertension and diabetes (9, 11, 12). Although a number of national programmes and policies have been introduced to curb the growing burden of NCDs, the awareness, treatment and control of hypertension and diabetes remains a major challenge.

Punjab and Haryana, two major northern Indian states reported a high prevalence of non-communicable diseases in the state-wide risk factor surveys (13, 14). The STEPS survey findings in both the state survey suggests that NCD risk factors are, in general, common and almost uniformly prevalent in the adult population of the two states. The fact that only 1% of the study population in Punjab and 0.2% of the study population in Haryana was found to be free of all studied NCD risk factors is an indication of growing epidemic of NCDs in the two states. The cascade care for NCDs, that is, the proportion of people with related conditions who have been screened, know their diagnosis, are taking medications, and their condition is under control is a useful concept that provides information to design intervention and to evaluate the health system performance. To date, few large-scale population based studies have been conducted in India, reporting the prevalence and control rates of hypertension and diabetes and the point at which people are lost from care (15). To fill the knowledge gap, awareness, treatment, and control of diabetes and hypertension were studied among the adult population in these two major northern Indian states.

## Methodology

### Study Design

The data used for the study is from the state-wide NCD risk factors surveys were conducted in Punjab and Haryana, India using the WHO STEPS methodology. The data is state representative and the survey were conducted in 2014–15 and 2016–17, respectively. The detailed methodology of the surveys has been published separately (13, 14). Standardized questionnaire was used to determine the behavioral risk factors in step one followed by anthropometric measurements for physical risk factors in step two and in the third step serum and urine samples were collected for biochemical risk factors.

### Sampling

This study reports the results from cross-sectional surveys conducted in two states using a multi-stage stratified sample. The subjects of the survey were 18–69 year-old adult men and women living in urban and rural areas. The estimated value of the total sample size of the two surveys was calculated by summing up the age, gender, and residential strata and adjusting them to a design effect of 1.5 (16). The sample sizes for Punjab 5400 and Haryana 5250 were calculated after considering the response rate of 90%.

The primary sampling units (PSUs) were villages in rural areas and census enumeration blocks in urban areas. In both urban and rural areas, one individual was selected from the selected household following the KISH method (17). The WHO STEPS questionnaire version 3.1 (18), with local adaptations was used in both the surveys.

### Data Collection

In both the surveys, socio-demographic and behavioral information was collected in STEP 1. The information on tobacco and alcohol use, health screening, history of chronic conditions and family history of NCDs was collected. For STEP 2, physical measurements such as height, weight, waist circumference, and blood pressure were collected using standard procedures and protocols. The instruments were calibrated periodically during the survey. The standard procedures of measurement have been described in detail previously (13, 14). The blood pressure was measured in sitting position on the right arm supported at the level of the heart using calibrated electronic equipment (OMRON HEM 7120, Omron Cooperation, Japan) (19). Total of three measurements were recorded at 2 min interval each and for the analysis the average of last two readings were taken. STEP 3, i.e., the biochemical measurements were conducted on serum samples to assess fasting blood glucose STEP 3 was done on a subsample of the total participants. Alternate individuals (50% of the total) were given written instructions regarding fasting and appointment date for blood test. Blood glucose was measured using finger prick blood samples and blood glucose measurement device (Optium H Freestyle) (20).

### Operational Definitions

Both surveys used cut-off values recommended in the WHO STEPS approach (18). Current tobacco use was defined as those who smoked and consumed smokeless tobacco in the past 30 days and current alcohol use as those who had consumed alcohol in the last 1 year. Obesity was defined as BMI 27.5 kg/m^2^ which is the standard cut-off for Asian population (21). Hypertension was defined as systolic blood pressure (SBP) ≥ 140 mm of Hg, or diastolic blood pressure (DBP) ≥ 90 mm of Hg or the use of blood pressure-lowering medications for hypertension. Individuals with fasting capillary blood glucose of ≥126 mg/dl or on medications for high blood sugar were considered to have diabetes mellitus. Individual was considered on treatment with current use of antihypertensive or antidiabetic medication. Controlled hypertension or diabetes was defined for those taking medication for the management of high BP or high blood glucose at the time of the interview and having systolic BP < 140 mmHg and diastolic BP < 90 mmHg and blood glucose < 126 mg/dl.

### Ethical Considerations

The study was approved by the Ethics Committee of the Postgraduate Institute of Medical Education and Research, Chandigarh. The technical advisory committee of both the surveys approved the research plan and supervised the implementation and execution of the two surveys. Written consent of all survey participants has been obtained. Complete privacy and confidentiality of participants was ensured.

### Statistical Analysis

Frequencies (percentages) or means and standard deviations were used to summarize the socio-demographic characteristics, physical measurements, and hypertension and diabetes status of the study participants. The primary outcome variables were awareness, treatment, and control of hypertension and diabetes The strength of associations of socio-demographic associated factors of hypertension and diabetes, awareness, treatment, and control were assessed by Odds-Ratios (OR) estimated in logistic regression models All estimates are presented with 95% confidence intervals (CIs), significance of difference in results between different groups was observed by comparing CIs. The associations were assessed using multivariate models adjusted for the covariates age, gender, residence, education, family history of hypertension and diabetes, current tobacco, and current alcohol status. Significance level was set at *p* < 0.05 for all hypothesis tests. SPSS version 21 (22) was used as the statistical software for analysis.

## Results

### Socio-Demographic and Behavioral Characteristics

Table 1 gives the socio-demographic and the behavioral characteristics of the study population of the two surveys in Punjab and Haryana. The surveys included a total of 5,127 participants in Punjab and 5,078 participants in Haryana. In Punjab, 54% women and 46% men stratified by age group, sex and place of residence were included. In Haryana, 55% of the total participants were females and 45% were males. Sixty-eight percent of the study sample in Haryana and 65% in Punjab belonged to 18–44 years age group. About 40% of the Punjab population and 44% of the Haryana population have never had their blood pressure measured. In the case of diabetes, the numbers are even higher, as 64% of the Punjab population and 74% of the Haryana population have never had their blood glucose measured. In Punjab, prevalence of current tobacco and alcohol use was 11.3 and 14.5%, respectively. While in Haryana, 23.5% were current tobacco users and 10.5% were current alcohol users.

**Table 1:** Socio-demographic and behavioral characteristics of the participants, Punjab and Haryana.

**Variable**		**Punjab (*N* = 5,127)**	**Haryana (*N* = 5,078)**
		***N* (%)**	***N* (%)**
**Age**	18–44	3,344 (65)	3,473 (68)
	45–69	1,783 (35)	1,605 (32)
**Gender**	Male	2,381 (46)	2,294 (45)
	Female	2,746 (54)	2,784 (55)
**Residence**	Rural	3,096 (60)	3,368 (66)
	Urban	2,031 (40)	1,710 (34)
**Education**	No formal schooling	1,208 (24)	514 (12)
	Less than primary school	281 (6)	321 (8)
	Primary school completed	987 (19)	743 (17)
	Secondary school completed	760 (15)	878 (21)
	High school completed	1,373 (27)	1,113 (26)
	College/University completed	348 (7)	575 (13)
	Post graduate degree	170 (3)	137 (3)
**Social group**	SC	1,927 (38)	1,742 (34)
	OBC/others	699 (14)	1,223 (24)
	General	2,410 (47)	2,082 (41)
	Refused	91 (1)	31 (1)
**Marital status**	Never married	838 (16)	612 (12)
	Currently married	3,875 (76)	4,132 (81)
	Separated	37 (1)	8 (0)
	Divorced	25 (1)	5 (0)
	Widowed	318 (6)	271 (5)
	Refused	35 (0)	50 (1)
**Measurement of blood pressure**	Never measured	1,998 (39)	2,209 (44)
**Measurement of blood sugar**	Never measured	3,316 (64)	3,773 (74)
**Family history**	Hypertension	1,957 (38.3)	2,162 (42.6)
	Diabetes	1,174 (23.2)	1,052 (20.8)
**Hypertension status**	Prevalence	2,030 (40.3)	1,371 (26.2)
	Aware	980 (48.3)	457 (33.3)
	On treatment	611 (30.1)	361 (26.3)
	Controlled	373 (18.3)	164 (12.0)
		***N*** **=** 2,366^*^	***N*** **=** 2,488^*^
**Diabetes status**	Prevalence	336 (14.2)	348 (15.5)
	Aware	115 (34.2)	103 (29.5)
	On treatment	95 (28.2)	78 (22.4)
	Controlled	48 (14.2)	48 (13.7)

* *Blood glucose was measured on a subsample of the total participants*.

### Prevalence, Awareness, Treatment, and Control of Hypertension

**Figure 1:**
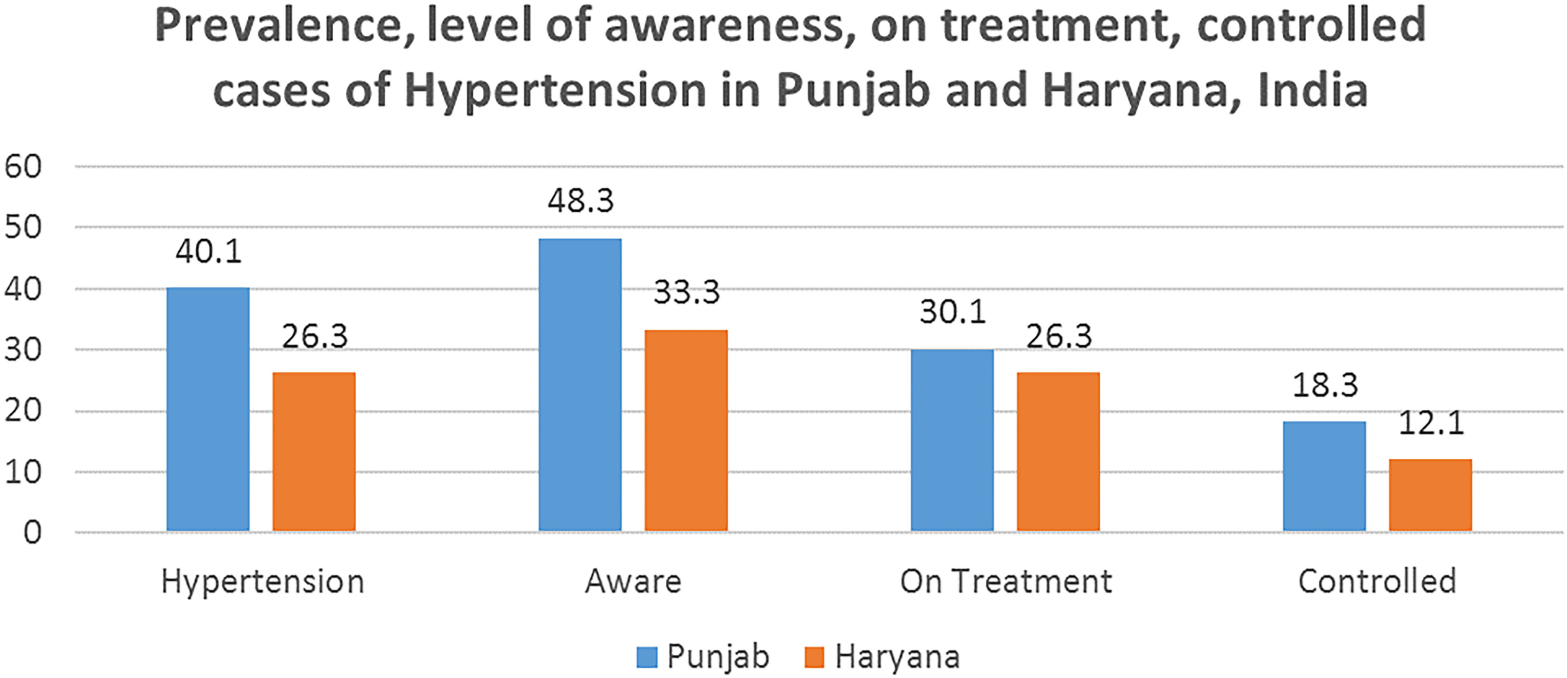
Extent of awareness, on treatment, controlled cases of hypertension in Punjab and Haryana, India.

Figure 1 shows the extent of awareness, treatment and control of hypertensive cases in Punjab and Haryana. The prevalence of hypertension in Punjab was 40.1% while that in Haryana was 26.2%. In Punjab, only 48.3% of the hypertensive were aware of their condition, 30.9% are on treatment while only 18.3% of the cases were controlled. While in Haryana 33.4% of the respondents were aware of their condition, 26.3% are on treatment while only 12% of the cases are controlled. The prevalence of hypertension was found to be higher among males in both the states, i.e., 47.4% in Punjab and 29.5% in Haryana. Further analysis shows that more females were on treatment in both the states in comparison to men. Also, in both the states, higher proportion the urban population were on treatment than the rural population (Table 2).

**Table 2:** Proportion (%) of hypertension awareness, treatment, and control in Punjab and Haryana, India.

**Demographic variables**	**Punjab**, ***n*** **(%)**				**Haryana**, ***n*** **(%)**			
	**Hypertensnsives (*N* = 5,027)**	**Aware (*N* = 2,030)**	**On treatment (*N* = 2,030)**	**Controlled (*N* = 2,030)**	**Hypertensives (*N* = 5,078)**	**Aware (*N* = 1,371)**	**On treatment (*N* = 1,371)**	**Controlled (*N* = 1,371)**
**Gender**								
Male	1,093 (47.4)	324 (29.6)	197 (18.1)	100 (9.1)	719 (29.5)	268 (37.2)	121 (16.9)	70 (9.8)
Female	937 (31.5)	656 (70.4)	414 (44.1)	273 (25.3)	652 (22.1)	189 (28.9)	240 (36.8)	94 (14.4)
**Residence**								
Rural	1,225 (41.1)	590 (48.2)	347 (28.3)	203 (16.6)	851 (24.4)	209 (24.5)	213 (25.1)	106 (12.4)
Urban	805 (38.7)	390 (48.7)	264 (32.8)	170 (21.2)	520 (28.8)	248 (47.7)	148 (28.4)	58 (11.2)
**Age (years)**								
18–44	979 (30.4)	407 (41.6)	214 (21.9)	149 (15.2)	738 (20.7)	217 (29.4)	159 (21.5)	87 (11.8)
45–69	1,051 (60.6)	573 (54.6)	397 (37.8)	224 (21.3)	633 (39.1)	240 (37.9)	202 (44.2)	77 (12.2)

### Prevalence, Awareness, Treatment, and Control of Diabetes

**Figure 2:**
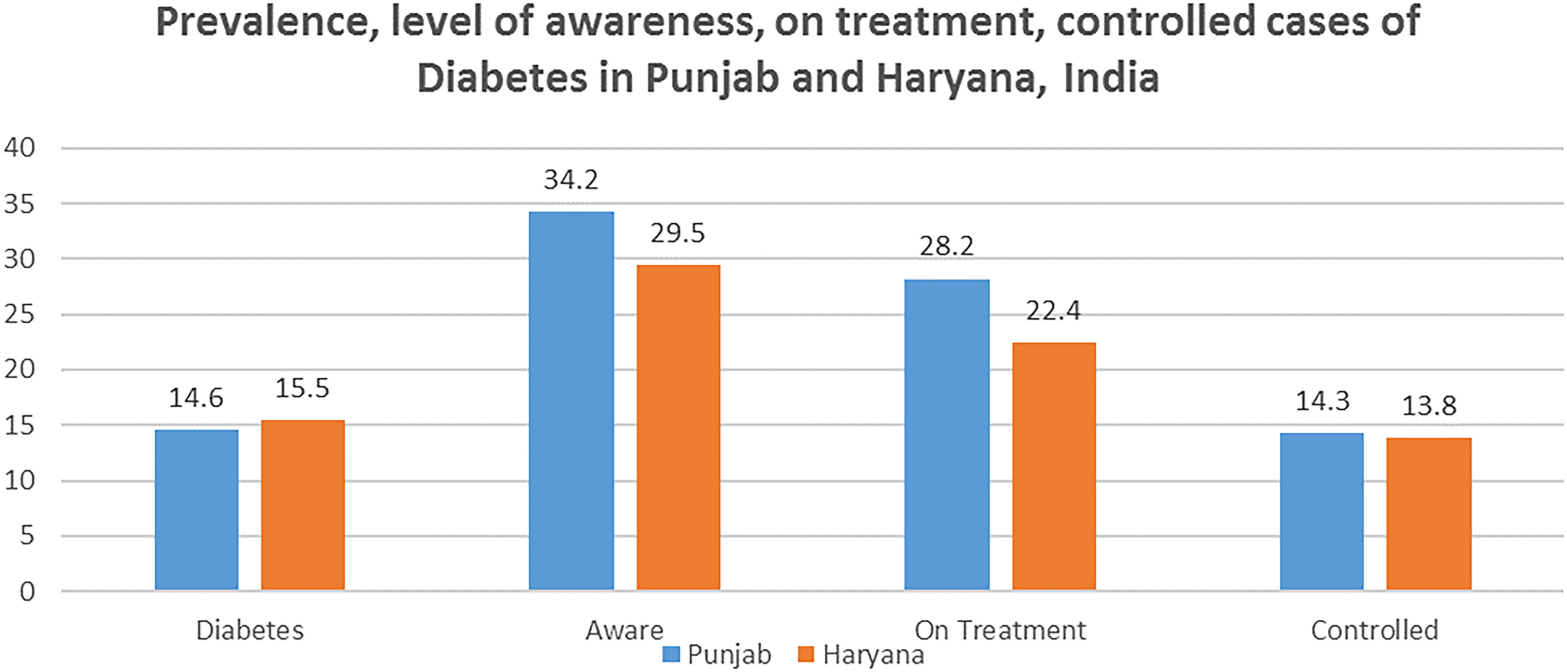
Extent of awareness, on treatment, controlled cases of diabetes in Punjab and Haryana, India.

The prevalence of diabetes was 14.3 and 15.1% in Punjab and Haryana, respectively. In Punjab 34.2% of diabetics aware of their condition, 28.2% are on treatment while only 14.2% of the cases are controlled. Figure 2 highlights the awareness and control rates in Haryana were similar to that in Punjab. 29.5% of the respondents were aware of their condition, 22.4% are on treatment while only 13.8% of the cases of diabetes were controlled. Table 3 highlights that the prevalence of diabetes was higher in rural areas in both Punjab and Haryana. Furthermore, in both the states, higher proportion the urban population were on treatment than the rural population.

**Table 3:** Proportion (%) of diabetes awareness, treatment, and control in Punjab and Haryana, India.

**Demographic Variables**	**Punjab**, ***n*** **(%)**				**Haryana**, ***n*** **(%)**			
	**(*****N*** **=** **2,366)**				**(*****N*** **=** **2,488)**			
	**Diabetics (*N* = 2,366)**	**Aware (*N* = 336)**	**On treatment (*N* = 336)**	**Controlled (*N* = 336)**	**Diabetics (*N* = 2,488)**	**Aware (*N* = 348)**	**On treatment (*N* = 348)**	**Controlled (*N* = 348)**
**Gender**
Male	133 (14.0)	42 (28.6)	50 (44.1)	33 (25.1)	140 (14.2)	62 (33.2)	38 (16.9)	14 (9.8)
Female	203 (14.6)	73 (38.4)	45 (38.1)	15 (9.3)	208 (17.6)	41 (26.9)	40 (28.8)	34 (13.4)
**Residence**
Rural	146 (14.0)	39 (48.2)	347 (24.3)	203 (12.6)	189 (12.6)	54 (24.5)	32 (25.1)	26 (9.4)
Urban	190 (14.6)	76 (30.7)	264 (30.8)	170 (16.2)	159 (19.7)	49 (35.7)	46 (28.4)	22 (15.2)
**Age (years)**
18–44	98 (8.4)	407 (41.6)	214 (21.9)	149 (15.2)	165 (15.0)	59 (26.4)	27 (21.5)	12 (10.8)
45–69	238 (27.7)	573 (54.6)	397 (37.8)	224 (21.3)	183 (21.3)	44 (35.9)	51 (34.2)	36 (14.2)

### Factors Associated With Awareness, Treatment, and Control of Hypertension

Table 4 presents findings from multiple logistic regression that identified factors associated with hypertension awareness, treatment, and control. It highlights that the family history of hypertension is associated with higher odds of being aware, and controlled blood pressure levels in Punjab with OR of 1.1 (95% CI, 0.6–1.8) and OR 0.1 (95% CI, 0.7–1.4). While in Haryana people with family history of hypertension were more likely to be aware, on treatment and had better control rates for hypertension with OR 2.49 (95% CI, 2.3–2.6), 1.6 (95% CI, 1.4–2.8), 2.0 (95% CI, 1.4–2.7)/ No formal schooling was negatively associated with hypertension awareness, treatment, and control in both the states. Also, the current tobacco and alcohol use is related with poor control status of the blood pressure levels while higher awareness and treatment rates in both states.

**Table 4:** Multivariable Logistic Regression Model to assess Factors associated with awareness, treatment, and control of hypertension in Punjab and Haryana, India.

		**Punjab**			**Haryana**		
		**Awareness**	**Treatment**	**Control**	**Awareness**	**Treatment**	**Control**
		**OR (95% CI)**	**OR (95% CI)**	**OR (95% CI)**	**OR (95% CI)**	**OR (95% CI)**	**OR (95% CI)**
Education	No formal schooling	0.3 (0.04–1.3)	0.7 (0.3–1.0)	0.3 (0.1–0.6)	0.94 (0.6–1.4)	0.9 (0.5–1.8)	0.4 (0.2–0.9)
	Primary education	0.5 (0.4–1.8)	0.7 (0.4–0.9)	0.8 (0.4–1.2)	1.1 (0.7–1.6)	1.0 (0.8–3.0)	0.7 (0.3–1.1)
	Secondary education	0.6 (0.4–1.9)	0.9 (0.4–1.8)	1 (06–1.6)	0.8 (0.6–1.3)	1.3 (0.7–2.3)	0.8 (0.5–1.4)
	Higher education	1	1	1	1	1	1
Current tobacco use	Yes	1.7^**^ (1.1–2.5)	0.8^*^ (0.6–1.1)	0.9 (0.6–1.7)	1.8^**^ (1.2–2.4)	1.2 (0.7–1.9)	0.8 (0.5–1.3)
	No	1	1	1	1	1	1
Current alcohol use	Yes	0.9 (0.6–1.1)	0.7 (0.5–1.0)	0.8 (0.4–1.2)	0.5^*^ (0.3–0.7)	1.3 (0.7–2.2)	0.9 (0.7–1.8)
	No	1	1	1	1	1	1
Gender	Males	0.2^*^ (0.1–1.7)	0.4^*^ (0.2–0.9)	0.8 (0.4–1.4)	1.27 (0.85–1.9)	0.7^*^ (0.4–1.2)	0.5^*^ (0.3–0.7)
	Females	1	1	1	1	1	1
Age group	18–44	3.6^*^ (3.1–4.1)	1.2 (0.8–1.9)	2.3 (1.8–3.1)	3.2^*^ (2.5–4.1)	1.4 (1.1–2.0)	0.4^*^ (0.3–0.7)
	45–69	1	1	1	1	1	1
Family history of hypertension	Yes	1.1^*^ (0.6–1.8)	0.8 (0.6–0.9)	1.1 (0.7–1.5)	2.49^**^ (2.3–2.6)	1.6^*^ (1.4–2.8)	2.0^**^ (1.4–2.7)
	No	1	1	1	1	1	1
Residence	Rural	1.0 (0.8–1.2)	0.9 (0.5–1.4)	0.8 (0.8–1.4)	1.08 (0.8–1.4)	0.9 (0.7–1.3)	1.1 (0.7–1.3)
	Urban	1	1	1	1	1	1

* *p < 0.05*;

** *p < 0.001*.

*The variables adjusted include education, residence, age group, family history of hypertension, gender, current tobacco, and current alcohol use*.

### Factors Associated With Awareness, Treatment, and Control of Diabetes

The multiple logistic regression analysis to find the factors associated with awareness, treatment and control rates of diabetes are given in Table 5. Men were more likely to be aware of their diabetes status than women in Punjab and Haryana (OR = 1.1, 95% CI, 0.8–1.9; OR: 1.27, 95% CI, 0.85–1.9).The analysis highlights that the family history of diabetes is associated with higher odds of being aware, treatment, and controlled blood glucose levels in both Punjab and Haryana. Also, the current tobacco and alcohol use is negatively related with control status of the blood glucose levels.

**Table 5:** Multivariable Logistic Regression Model to assess Factors associated with awareness, treatment, and control of diabetes in Punjab and Haryana, India.

		**Punjab (*****N*** **=** **2,366)**			**Haryana (*****N*** **=** **2,488)**		
		**Awareness**	**Treatment**	**Control**	**Awareness**	**Treatment**	**Control**
		**OR (95% CI)**	**OR (95% CI)**	**OR (95% CI)**	**OR (95% CI)**	**OR (95% CI)**	**OR (95% CI)**
Education	No formal schooling	0.6 (0.6–1.1)	0.1^*^ (0.03–0.4)	0.8 (0.3–1.8)	0.6 (0.4–1.4)	0.8 (0.5–1.8)	0.4 (0.2–0.9)
	Primary education	0.8 (0.7–1.4)	0.3 (0.05–0.8)	0.7 (0.6–2.6)	0.4 (0.3–1.6)	0.8 (0.8–3.0)	0.7 (0.3–1.1)
	Secondary education	0.4 (0.4–1.0)	0.8 (0.3–1.2)	0.9 (0.4–1.9)	0.8 (0.6–1.3)	1.3 (0.7–2.3)	0.9 (0.5–1.4)
	Higher education	1	1	1	1	1	1
Current tobacco use	Yes	1.04 (0.6–1.9)	0.9 (0.7–1.8)	0.7 (0.6–2.0)	0.8^**^ (0.6–2.4)	1.2 (0.7–1.9)	0.6 (0.5–1.3)
	No	1	1	1	1	1	1
Current alcohol use	Yes	1.3 (0.9–1.5)	0.8 (0.4–1.1)	0.4 (0.3–1.3)	0.5^*^ (0.3–0.7)	1.3 (0.7–2.2)	0.7 (0.7–1.8)
	No	1	1	1	1	1	1
Gender	Males	1.1 (0.8–1.9)	1.2 (0.8–2.0)	1.09 (0.4–2.0)	1.27 (0.85–1.9)	0.9 (0.4–1.4)	1.5^*^ (0.3–1.7)
	Females	1	1	1	1	1	1
Age group	18–44	0.2^*^ (0.2–0.4)	3.0^*^ (1.3–4.0)	2.3 (1.4–3.2)	3.2^*^ (2.5–4.1)	1.4 (1.1–2.0)	0.4^*^ (0.3–0.7)
	45–69	1	1	1	1	1	1
Family history of Diabetes	Yes	1.2^*^ (0.9–1.5)	1.1^*^ (0.5–2.5)	0.5 (0.2–1.2)	1.49^**^ (1.3–2.7)	1.6^*^ (1.4–2.8)	0.6^**^ (0.4–2.7)
	No	1	1	1	1	1	1
Residence	Rural	0.8 (0.6–1.1)	1.5 (0.6–2.2)	0.9 (0.4–1.9)	1.0 (0.8–1.4)	0.9 (0.7–1.3)	1.1 (0.7–1.3)
	Urban	1	1	1	1	1	1

* *p < 0.05*;

** *p < 0.001*.

*The variables adjusted include education, residence, age group, family history of diabetes, gender, current tobacco, and current alcohol status*.

## Discussion

The study reports a high prevalence of hypertension and diabetes in Punjab and Haryana, the two major north Indian states. The estimates for diabetes, i.e., 14 and 15.5% are higher than the global average of 9% and that of other South Asian countries (23, 24). The prevalence of hypertension reported in Punjab is also higher than the national average of 29.8% (25). Economic development, changes in lifestyle and diet, and an increase in life expectancy may explain the rapidly increasing prevalence of hypertension and diabetes in developing countries (26).

A large proportion of the population in these two states suffers from uncontrolled high blood pressure and diabetes. Eighteen percent of cases of hypertension in Punjab, while only 12% of cases of hypertension in Haryana were under control, which is startling. The results of these two states are consistent with other studies conducted in different states of India. Several previous studies conducted in India reported similar data on blood pressure and diabetes control rates which were alarmingly low. The level of awareness ranges from 26 to 59%, while the control rates are as low as 10–45% (12, 25, 27–32).

A systematic review of population-based studies from 90 countries conducted in 2016, showed that less than half (46.5%) of adults with hypertension were aware of their condition, 36.9% were treated with antihypertensive medication, and only 13.8% had their blood pressure controlled worldwide (9). High-income countries had almost double the proportions of awareness (67.0 vs. 37.9%) and treatment (55.6 vs. 29.0%) and 4 times the proportion of control among patients with hypertension (28.4 vs. 7.7%) in comparison with low- and middle income countries.

The country-level analysis of the hypertension care cascade, however, disguises the large variation in the care cascades among states and population groups (25). In the studies conducted abroad, the percentage of people who were aware of their hypertensive status varied between 24.1 and 81.8%. The proportion of treated population among the aware population was ranged from 36.1 to 82.1%. The percentage of controlled disease varied between 10.6 and 50.7% of the treated population (33–36).

The study also highlights that having a family history of hypertension or diabetes is a predictor for awareness and treatment for the condition. Implications of our findings are that there needs to be substantial investment in health promotion to raise awareness, and changes in healthcare delivery that address treatment and control of diabetes regardless of socio-demographic status.

Hypertension and diabetes are a major public health problem in Punjab and Haryana and awareness, treatment, and control rates are low which require specific interventions with a focus on access to treatment, regular follow up for better control. Despite the screening process which have been undertaken for NCDs including hypertension diabetes being a major component of the National Programme for Control of Cancer, Diabetes, Cardiovascular Diseases, and Stroke (NPCDCS) in India, implementation is abysmal (37, 38). The low levels of awareness and control rates indicate toward the need for aggressive health promotion program under NPCDCS.

The undiagnosed cases of hypertension and diabetes if left untreated are more prone to complications and morbidity including cardiac failure, kidney failure, cerebral stroke, and damage to blood vessels (39, 40). Hence, it is imperative to identify and offer early therapy to these individuals and ensure regular follow up.

At the primary care level, screening for these conditions among asymptomatic persons shall be useful in their early diagnosis and management. Appropriate counseling to improve adherence to treatment and advice is likely to result in better control of these conditions. While improvements are needed along the entire hypertension and diabetes care cascade, this highlights a particular need for interventions that focus on the awareness and treatment steps of the cascade. There is an urgent need to effectively implement the existing national NCD programmes in these states in India.

### Strengths and Limitations of the Study

The strengths of the present study are that it utilizes the data from two state wide representative population based, multistage sample including adults 18–69 years of age. Both the surveys had a high response rate and followed the standard WHO STEPS methodology. One of the limitation of the surveys was drawing the blood sample from a subset of the total population owing to the resource constraints. Also, the blood glucose was measured was through a glucometer instead of glucose measurement on venous blood sample.

## Data Availability Statement

The datasets presented in this study can be found in online repositories. The names of the repository/repositories and accession number(s) can be found below: https://doi.org/10.1371/journal.pone.0208872.s005; https://doi.org/10.1371/journal.pone.0157705.s001.

## Ethics Statement

The studies involving human participants were reviewed and approved by the Institute Ethics Committee, Postgraduate Institute of Medical Education and Research. The patients/participants provided their written informed consent to participate in this study.

## Author Contributions

JT: conceptualization, funding acquisition, and resources. RN: data curation and writing – original draft. JT and RN: formal analysis, investigation, methodology, validation, and writing – review and editing. All authors contributed to the article and approved the submitted version.

## Funding

This study was supported by two state-wide surveys of National Health Mission (State NCD Cell, Punjab and Haryana), Ministry of Health and Family Welfare, Government of India.

## Conflict of Interest

The authors declare that the research was conducted in the absence of any commercial or financial relationships that could be construed as a potential conflict of interest.

## Publisher's Note

All claims expressed in this article are solely those of the authors and do not necessarily represent those of their affiliated organizations, or those of the publisher, the editors and the reviewers. Any product that may be evaluated in this article, or claim that may be made by its manufacturer, is not guaranteed or endorsed by the publisher.
